# Immunomodulatory of sesquiterpenoids and sesquiterpenoid dimers-based toll-like receptor 4 (TLR4) from *Dysoxylum parasiticum* stem bark

**DOI:** 10.1038/s41598-024-65829-0

**Published:** 2024-07-06

**Authors:** Al Arofatus Naini, Tri Mayanti, Erina Hilmayanti, Xuhao Huang, Kazuya Kabayama, Atsushi Shimoyama, Yoshiyuki Manabe, Koichi Fukase, Unang Supratman

**Affiliations:** 1 Department of Chemistry, Faculty of Mathematics and Natural Sciences, Universitas Padjadjaran, Jatinangor, Sumedang, West Java 45363 Indonesia; 2 Central Laboratory, Universitas Padjadjaran, Jatinangor, Sumedang, West Java 45363 Indonesia; 3 Faculty of Mathematics and Natural Sciences, Study Centre of Natural Product Chemistry and Synthesis, Univers itas Padjadjaran, Jatinangor, Sumedang, West Java 45363 Indonesia; 4 Department of Chemistry, Graduate School of Science, Osaka University, 1-1 Machikaneyama-Cho, Toyonaka, Osaka 560-0043 Japan; 5 Institute for Radiation Sciences, Osaka University, 1-1 Machikaneyama-Cho, Toyonaka, Osaka 560-0043 Japan

**Keywords:** *Dysoxylum parasiticum* (Osbeck) Kosterm., Sesquiterpenoids and sesquiterpenoid dimers, Immunomodulatory TLR4, Proinflammatory cytokines, Chemical biology, Drug discovery

## Abstract

In recent decades, the interest in natural products with immunomodulatory properties has increased due to their therapeutic potential. These products have a wider range of pharmacological activities and demonstrate lower toxicity levels when compared to their synthetic counterparts. Therefore, this study aimed to investigate the immunomodulatory effects of sesquiterpenoids (SQs) and sesquiterpenoid dimers (SQDs) isolated from *Dysoxylum parasiticum* (Osbeck) Kosterm. stem bark on human and murine cells, particularly focusing on toll-like receptor 4 (TLR4). Utilizing the secreted alkaline phosphatase (SEAP) assay on engineered human and murine TLR4 of HEK-Blue cells, antagonist TLR4 compounds were identified, including SQs **6**,** 9**, and **10**, as well as SQDs **17** and **22**. The results showed that 10-hydroxyl-15-oxo-*α*-cadinol (9) had a potent ability to reduce TLR4 activation induced by LPS stimulation, with minimal toxicity observed in both human and murine cells. The SEAP assay also revealed diverse immune regulatory effects for the same ligand. For instance, SQs **12**,** 14**, and **16** transitioned from antagonism on human to murine TLR4. The SQs (**4**,** 7**,** 11**, and **15**) and SQDs (**18–20**) offered partial antagonist effect exclusively on murine TLR4. Furthermore, these selected SQs and SQDs were assessed for their influence on the production of proinflammatory cytokines TNF-*α*, IL-1*α*, IL-1*β*, and IL-6 of the NF-κB signaling pathway in human and murine macrophage cell lines, showing a dose-dependent manner. Additionally, a brief discussion on the structure-activity relationship was presented.

## Introduction

The urgent need for the discovery of new therapeutic candidates is indicated by the unceasing emergence of immune health issues^1,2^. Recently, there has been a huge interest in the utilization of immunomodulation drugs derived from natural products due to significant efficacy and minimal negative side effects^3,4^. Toll-like receptors (TLRs) play a crucial role in recognizing conservative molecules of pathogenic microorganisms such as lipopolysaccharide (LPS), lipid A, and lipopolysaccharide (LOS) from Gram-negative bacteria^5–7^. Among these receptors, TLR4 is the first member and is mainly secreted on monocytes, macrophages, dendritic cells, and hematopoietic cells^8,9^. Through a multistep molecular recognition process, TLR4 responds quickly to minute amounts of circulating LPS. This process begins with the transfer of LPS monomers from aggregates in solution to LPS-binding protein (LPB), thereby causing the protein to cluster to differentiation 14 (CD14) and myeloid differentiation factor 2 (MD-2)^10^. The manipulation of immune responses through TLR4-mediated pathways shows promise as a pharmacological intervention approach^11^. These strongly suggest that TLR4 regulation is a convincing target for therapeutic control of a wide range immunomodulatory-based diseases. Meanwhile, secondary metabolites such as terpenoids, flavonoids, and alkaloids promote alternative immunomodulating properties, specifically as anti-inflammatory agent^12^. Sesquiterpenoid derivatives, constituting the most diverse subclass of the terpenoid group, are not only distinguished by their fascinating structures but are also intriguing in their bioactivities^13^. This has prompted the exploration of the potential of the derivatives as new lead compound in promoting health, particularly in immunomodulation^12,14^. The Meliaceae family, a widely recognized medicinal plant discovered in tropical and subtropical regions, comprises the extensive genus *Dysoxylum*, which consists of over 200 species. Its origins span across Australia, New Zealand, India, Southeast Asia, and Indonesia^15–17^. Numerous species of this genus are used in traditional medicine against skin irritations, digestive ailments, antiperiodic, and emmenagogue properties^15,18^. Previous studies on the species reported various phytoconstituents with anti-inflammatory activities, including sesquiterpenoids^19,20^, while their potential as immunostimulants shows great activation yet remains unexplored^21^.

*Dysoxylum parasiticum* (Osbeck) Kosterm. (Meliaceae) (http://powo.science.kew.org/taxon/578268-1/) is a species endemic to Indonesia and hold significance in spiritual activity among the Bali people. The species is popularly known as “Majegau” or divine tree due to its characteristic fragrances. The pioneer phytochemical investigations of this plant carried out by our group afforded 16 sesquiterpenoids (SQs) and six sesquiterpenoid dimers (SQDs), involving four SQs and six SQDs, first found from this plant^22–24^. Since the potential of all SQs and SQDs as immunomodulatory remains unexplored, this study aimed to investigate the immunomodulatory activity based on TLR4 receptors. The evaluation of TLR4 activation was performed against human and murine HEK-Blue cells using a secreted alkaline phosphate (SEAP) assay in the presence or absence of LPS. The SEAP will be induced by TLR4 stimulation on the cell membrane of the downstream transcript of NF-κB. Therefore, the level of SEAP in the cell supernatant relatively could represent the TLR4 and NF-κB activations. An enzyme-linked immunosorbent assay (ELISA) was further utilized to test the potential of SQs and SQDs to modulate TLR4 signaling pathways against THP-1 human monocytes and RAW 264.7 murine macrophages, focusing on proinflammatory cytokines TNF-*α*, IL-1*α*, IL-1*β*, and IL-6. The study also included an examination of their structure-activity relationship (SAR) to identify the crucial molecular component influencing their immunomodulatory effects.

## Results and discussions

**Figure 1:**
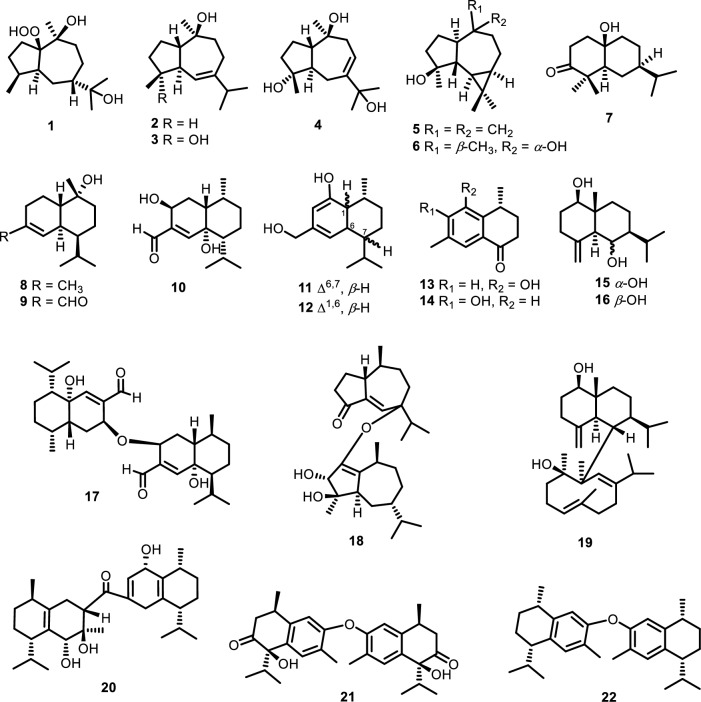
The structures of all isolated compounds **1–22**.

The phytochemical investigation of the *n-*hexane extract of *D. parasiticum* yielded sesquiterpenoids (SQs): 10*β*,11-dihydroxy-1*β*-hydroperoxide-4*αH*,5*αH*,7*βH*-guaiane (**1**), (1S*,4S*,5R*,10S*)-guai-6-ene-10*β*-ol (**2**), guaianediol (**3**), 4*α*,10*α*,11-trihydroxy-1*βH*,5*βH*-guai-7(8)-ene (**4**), spathulenol (**5**), 4*β*-10*α*-dihydroxyaromadendrane (**6**), 10*β*-hydroxy-4*α*,4*β*- dimethyl-5*αH*,7*αH*-eudesm-3-one (**7**), *α*-cadinol (**8**), 10-hydroxyl-15-oxo-*α*-cadinol (**9**), dysoticans A and B (**10**) and (**11**), 2,15-dihydroxycalamenene (**12**), schiffnerone B (**13**), aristegelone A (**14**), eudesm-4(15)-ene-1*β*,6*α*-diol (**15**), and eudesm-4(15)-ene-1*β*,6*β*-diol (**16**) and sesquiterpenoid dimers (SQDs): dysoticans C-H (**17–22**), including four SQs **1**,** 7**,** 10**, and **11** and six SQDs **17–22** that were first discovered from this plant (Fig. 1) after separation using normal and reverse techniques of column chromatography^22–24^. The potential immunomodulatory effect of 22 pure compounds was assessed in an experimental model of macrophage activation through the TLR4 signaling pathway. Various modulators have demonstrated distinct effects on human and murine TLR4. In certain cases, compounds showed a completely different from an antagonistic to agonistic effects when transitioning from human to murine TLR4/MD-2/CD-14 system^11^. In this study, the impact of SQ and SQD derivatives on human and murine macrophage cells by the stimulation of endotoxin lipopolysaccharide and without LPS, was examined. The results presented that among all the tested compounds (**1–22**), some had potent immunomodulatory effect on TLR4, including immunosuppressive/antagonist (**6**,** 9**,** 10**,** 22**) and partial immunosuppressive mTLR4 agents (**4**,** 7**,** 11**,** 15**,** 18**,** 19**,** 20**). Meanwhile, compounds **12**,** 14**, and **16** had an antagonistic effect on human TLR4, which completely switched to an agonistic effect on murine TLR4 (Table 1).

**Table 1:** The chemistry of isolated sesquiterpenoids (SQs) and sesquiterpenoid dimers (SQDs) along with their activity against human and murine TLR4.

Compounds	Type	Activity
10*β*,11-dihydroxy-1*β*-hydroperoxide-*4αH,5αH,7βH*-guaiane (**1**)	Guaiane	Inactive
(1S*,4S*,5R*,10S*)-guai-6-ene-10β-ol (**2**)		
Guaianediol (**3**)		
4*α*,10*α*,11-trihydroxy-1*βH*,5*βH*-guai-7(8)-ene (**4**)		Partial antagonist mTLR4
Spathulenol (**5**)	Aromadendrane	Inactive
4*β*-10*α*-dihydroxyaromadendrane (**6**)		Antagonist TLR4
10*β*-hydroxy-4*α*,4*β*-dimethyl-5*αH*,7*αH*-eudesm-3-one (**7**)	Eudesmane	
*α-*cadinol (**8**)	Cadinane	Inactive
10-hydroxyl-15-oxo-*α*-cadinol (**9**)		Partial antagonist TLR4
Dysotican A (**10**)		
Dysotican B (**11**)		Antagonist mTLR4
2,15-dihydroxycalamenene (**12**)		Contrasting effect
Schiffnerone B (**13**)	Tris-*nor-*cadinane	Inactive
Aristegelone A (**14**)		Contrasting effect
Eudesm-4(15)-ene-1*β*,6*α*-diol (**15**)	Eudesmane	Partial antagonist mTLR4
Eudesm-4(15)-ene-1*β*,6*β*-diol (**16**)		Contrasting effect
Dysotican C (**17**)	Asymmetrical cadinane-dimer	Antagonist TLR4
Dysotican D (**18**)	Asymmetrical guaiane-dimer	Partial antagonist mTLR4
Dysotican E (**19**)	Hybrid eudesmane/germacrene-dimer	
Dysotican F (**20**)	Asymmetrical cadinane-dimer	
Dysotican G (**21**)	Symmetrical cadinane-dimer	Inactive
Dysotican H (**22**)		Antagonist TLR4

### Modulation of SQs and SQDs on TLR4 signaling in HEK-Blue cells

**Figure 2:**
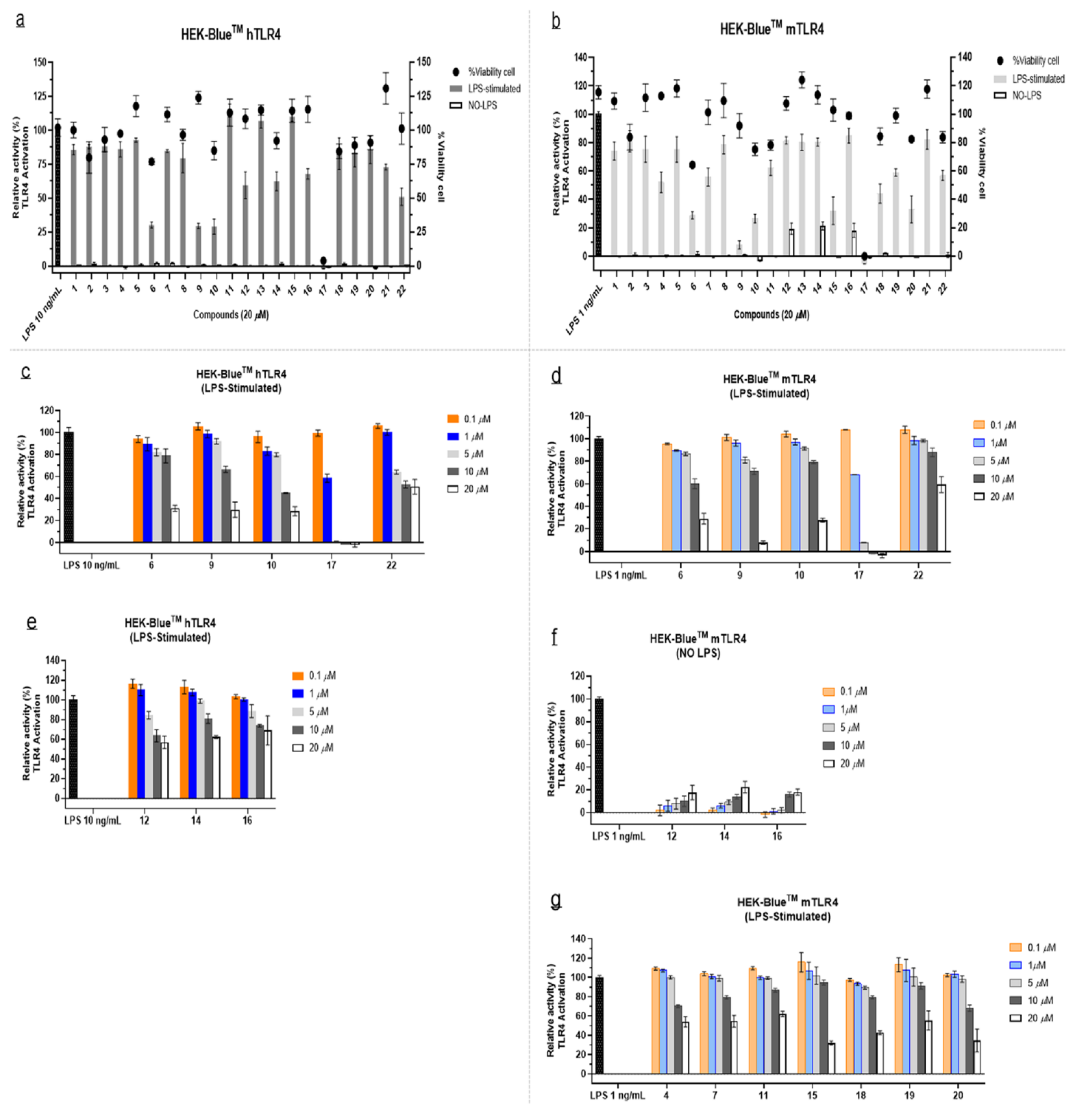
The SEAP assay and cytotoxicity of compounds **1–22** with the presence and absence of LPS against HEK-Blue™ hTLR4 (**a**) and HEK-Blue™ mTLR4 (**b**). The dose-dependent actions of the selected compounds with the antagonist (LPS-stimulated) on hTLR4 (**c**) and mTLR4 (**d**), switching effects with LPS-stimulated on hTLR4 (**e**) and NO LPS on mTLR4 (**f**), and partial antagonist (LPS-stimulated) on mTLR4 (**g**).

In order to evaluate the influence of isolated SQs and SQDs on the TLR4 signaling, we investigated the modulation of compounds **1–22** to antagonize TLR4 activation in LPS-stimulated cells, as well as examined their ability to act as TLR4 agonists in the absence of LPS. The immunomodulatory activity on a reporter gene cell line for human (HEK-Blue™ hTLR4) and a separate murine TLR4 (HEK-Blue™ mTLR4) was screened. These cells were engineered to stably express the human (hTLR4, hMD-2, and hCD14) and murine receptors (mTLR4, mMD-2, and mCD14) of the endotoxin recognition complex, as well as a SEAP reporter gene, respectively. The SEAP was controlled by NF-κB and AP-1 of TLR4-dependent transcription factors. Since assay optimization indicated saturation at 10 ng/mL on HEK-Blue mTLR4 cell (Fig. S1), the concentrations used in this experiment were 10 ng/mL and 1 ng/mL LPS on human and murine TLR4, respectively. As shown in Fig. 2a,b, SQs **6**,** 9**, and **10** and SQDs **17** and **22** inhibited signaling against HEK-Blue cells. The relative inhibition was lower than 60% of activation at a concentration of 20 μM by the presence of LPS, yielding a concentration-dependent response ranging from 0.1 to 20 μM (Fig. 2c,d). Compound **9**, identified as 10-hydroxyl-15-oxo-*α*-cadinol exhibited the strongest antagonist with relative inhibition of TLR4 activation ranging from 70 to 85% at concentration of 20 μM, without adversely affecting cell viability. Additionally, 4*β*-10*α*-dihydroxyaromadendrane (**6**) and another SQ, first originated from *D. parasiticum*, namely dysotican A (**10**) had strong antagonistic effect (70%) against these two cells and slightly showed lower cell viability (< 80%) against both HEK-Blue cells at 20 μM. The SQDs, first derived from this plant, particularly dysotican H (**22**) displayed moderate antagonistic with negligible toxicity to cells (> 80%) at 20 μM (Fig. 2a,b). Although dysotican C (**17**) showed toxicity effects at 10 μM, the evaluation in lower concentrations suggested that **17** moderately inhibited the LPS stimulation at 1 μM and strong inhibition at 5 μM without significant cytotoxicity, especially against a separate murine TLR4 (Fig. 2c,d, Figs. S2, S3). Several compounds performed inconsistency effects on human and murine TLR4 receptors. The SQs **12**,** 14**, and **16** switched from antagonism hTLR4 to agonism mTRL4, while **4**,** 7**,** 11**, and **15** as well as SQDs **18**,** 19**, and **20** presented partial antagonism against HEK-Blue mTLR4 (Fig. 2c–g). The viability assay confirmed that these compounds did not affect cell viability (Fig. 2a,b, Figs. S2, S3).

### The effect of potent SQs and SQDs on proinflammatory cytokines in human and murine TLR4 cells

Cytokines play an important role in innate immunity as mediators engaged in the elimination of invading foreign pathogens through the TLRs^25^. Macrophages contribute to enhancing both adaptive and innate immune responses^26^. Activated macrophages secrete proinflammatory cytokines, such as TNF-*α*, IL-1, and IL-6, leading to the induction of NF-κB proteins^27^. As the two HEK-Blue cells are transgenic cell lines, to further confirm the modulation effects of potent SQs and SQDs on human and murine TLR4, human monocyte THP-1 and murine macrophage RAW 264.7 cells served as in vitro models, using the natural TLR4 signaling system, by an ELISA experiment. Initially, the differentiation of the monocyte THP-1 cell lines into macrophage-like cells was performed by PMA-induction before compound treatment.

The effects of antagonists (**6**,** 9**,** 10**,** 17**,** 22**), contrasting effects (**12**,** 14**,** 16**), and partial antagonists (**4**,** 7**,** 11**,** 15**,** 18**,** 19**, and **20**) of SQs and SQDs on PMA-treated THP-1 and RAW 264.7 cells, were evaluated. These two cells were then exposed to different concentrations of SQ and SQD variants (0.1–20 μM) in the presence and absence of LPS for up to 24 h. Cell viability was tested after treatment by using a CCK-8 reagent. As shown in Figs. S4, S5, all the SQs and SQDs of interest did not show any cytotoxicity in THP-1 and RAW 264.7 cells even at a high concentration of 20 μM, except for **17**, which indicates toxicity at 10 μM. The three major proinflammatory cytokines (TNF-*α*, IL-1, and IL-6) were used to determine whether the potent SQs and SQDs isolated from the stem bark of *D. parasiticum* were engaged in the TLR4 activation. TNF-*α* and IL-1 are crucial cytokines in developing inflammation through IkB kinase (IKK) of the NF-κB signaling pathway, while IL-6 plays a positive role in inflammation through JAK/STAT signaling pathway.

**Figure 3:**
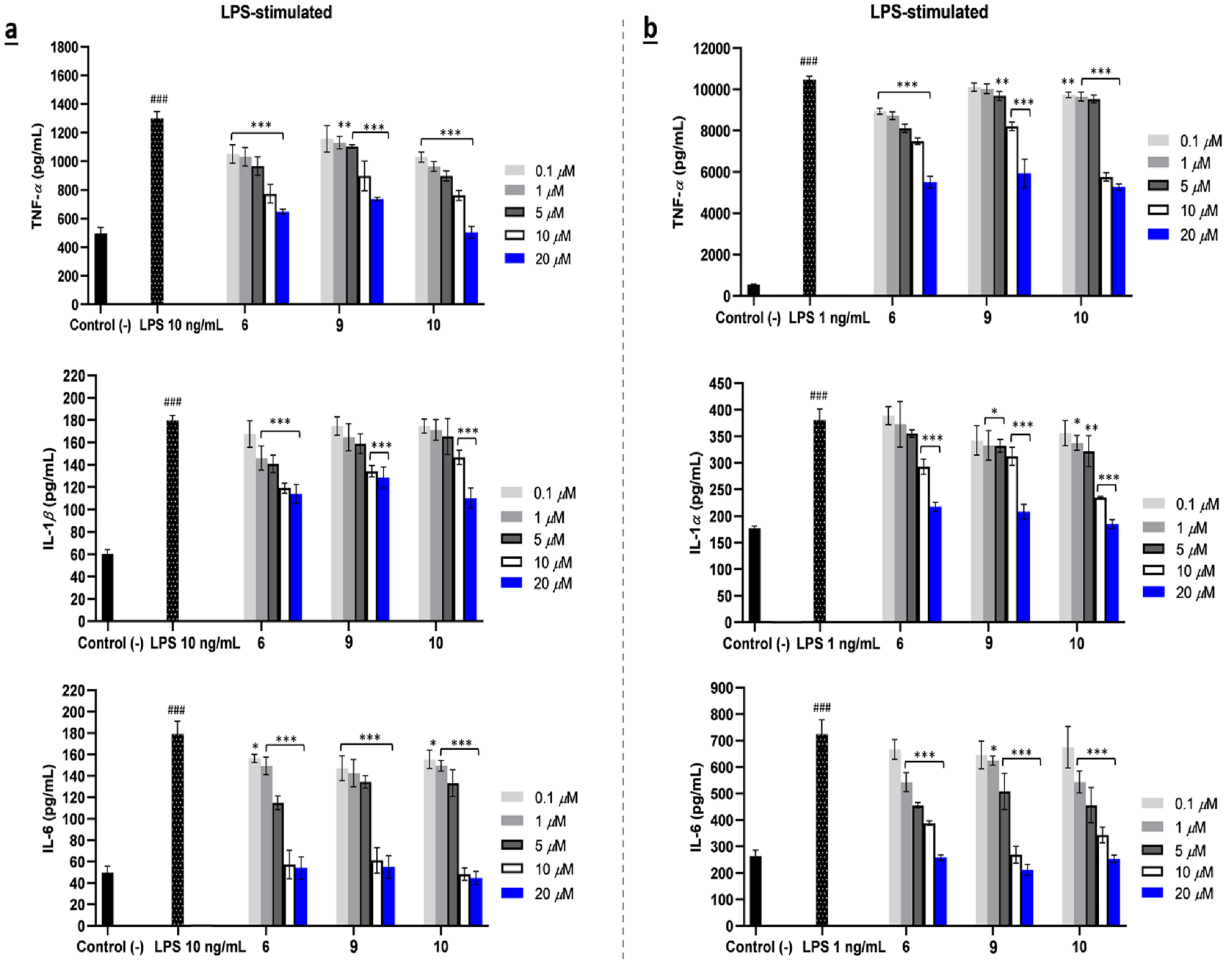
Proinflammatory cytokine productions of SQs (**6**,** 9**, and **10**) as antagonist TLR4 on LPS-stimulated of TNF-*α*, IL-1*β*, and IL-6 in THP-1 cells (**a**) and TNF-*α*, IL-1*α*, and IL-6 in RAW 264.7 cells (**b**). The results are shown as means ± SD (n = 4). Significant results are indicated as *p < 0.05, **p ≤ 0.01, ***p ≤ 0.001 compared to LPS and ^#^*p* < 0.05, ^##^*p* ≤ 0.01, ^###^*p* ≤ 0.001 compared to negative control.

**Figure 4:**
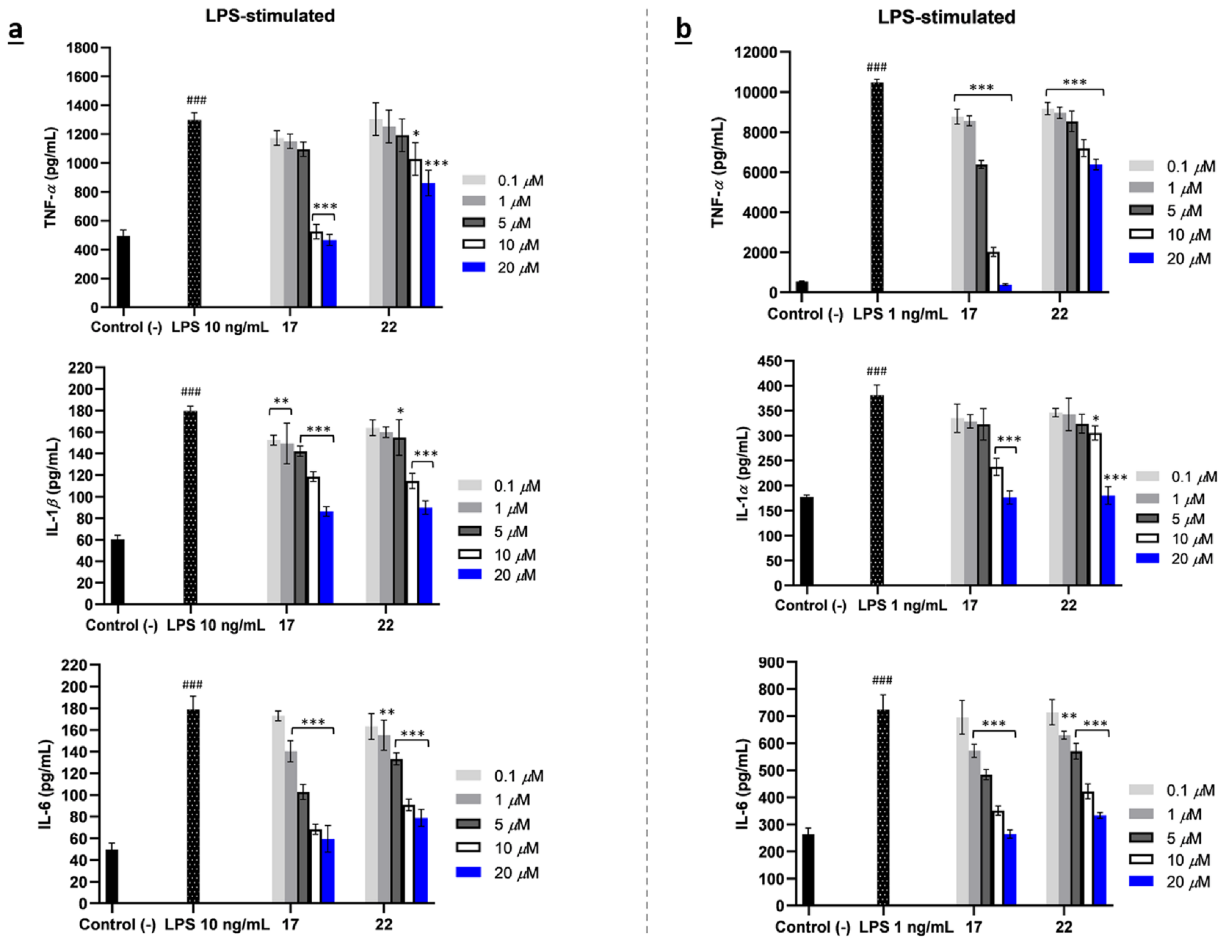
Proinflammatory cytokine productions of SQDs (**17** and **22**) as antagonist TLR4 on LPS-stimulated of TNF-*α*, IL-1*β*, and IL-6 in THP-1 cells (**a**) and TNF-*α*, IL-1*α*, and IL-6 in RAW 264.7 cells (**b**). The results are shown as means ± SD (n = 4). Significant results are indicated as *p < 0.05, **p ≤ 0.01, ***p ≤ 0.001 compared to LPS and ^#^*p* < 0.05, ^##^*p* ≤ 0.01, ^###^*p* ≤ 0.001 compared to negative control.

ELISA results revealed that the antagonist SQs (**6**,** 9**, and **10**) could significantly suppress IL-1*β* production in PMA-treated THP-1 cells. These three antagonists strongly suppressed the secretion of TNF-*α* and completely inhibited LPS simulation driven IL-6 at concentration of 10 μM, as shown in Fig. 3a. Similarly, in RAW 264.7 cells, antagonist SQs **6**,** 9**, and **10** had strong inhibition on TNF-*α* expression, as well as reduced the IL-6 cytokine to the same level as negative control at 20 μM in the presence of LPS 1 ng/mL. Additionally, the role of IL-1 signaling was investigated using IL-1*α* due to the murine RAW 264.7 being naturally deficient in a CARD domain (ASC) of apoptosis-associated speck-like protein, preventing the activation of pro-caspase-1 and the production of IL-1*β*^28^. The potential of SQ antagonists (**6**,** 9**, and **10**) as immunosuppressive agents was also complemented by the decrement of IL-1*α* level in murine TLR4 cells (Fig. 3b). In SQD antagonists, compounds **17** and **22** acted promising anti-inflammatory agents. Despite dysotican C (**17**) having cytotoxic effect in THP-1 and RAW 264.7 cell lines at 10 μM (Figs. S4, S5), it might still be used as immunosuppressive agent at a lower concentration. The considerable immunosuppressive activity of **17** was proved by the ELISA results, as demonstrated in Fig. 4b. The data showed that production of the three proinflammatory cytokines used against human and murine macrophage cells reduced when treated by 5 μM of **17**, specifically decreased the level of IL-1*β*, IL-6 in PMA-induced THP cells as well as TNF-*α*, IL-6 in RAW 264.7 cells. Furthermore, dysotican H (**22**) negatively modulated TLR4 signaling, promoting the three cytokines at 10 μM in a concentration dependent manner (Fig. 4a,b). These data clearly proved the potency of these SQ and SQD antagonists as negative regulators in human and murine cells, offering potential for anti-inflammatory drug discoveries. In particular, SQ dysotican A (**10**), and SQDs dysoticans C (**17**), H (**22**) represent compounds first isolated from this plant.

**Figure 5:**
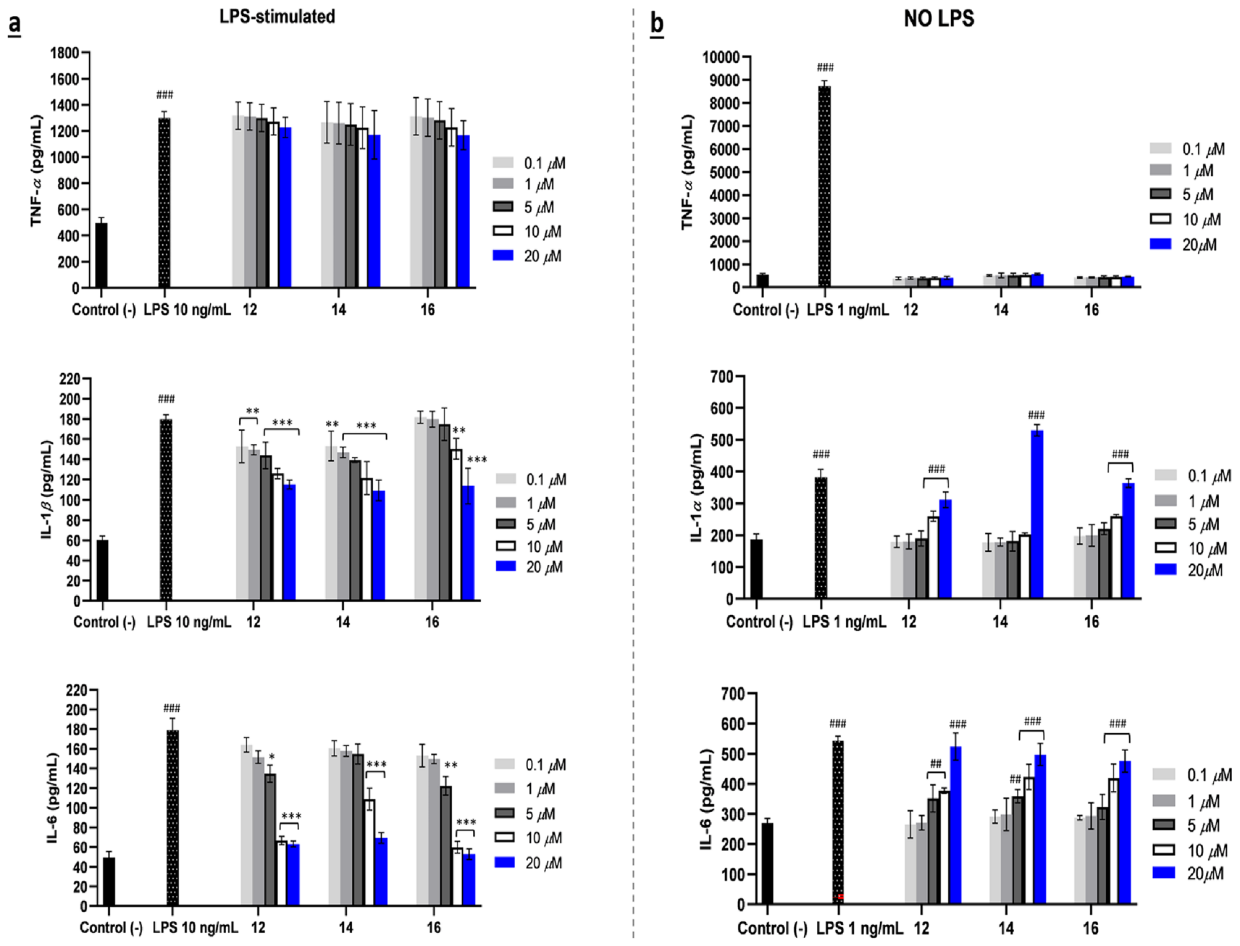
Proinflammatory cytokine productions of SQs (**12**, **14**, and **16**) with switching effects on LPS-stimulated of TNF-*α*, IL-1*β*, and IL-6 in THP-1 cells (**a**) and NO LPS of TNF-*α*, IL-1*α*, and IL-6 in RAW 264.7 cells (**b**). The results are shown as means ± SD (n = 4). Significant results are indicated as *p < 0.05, **p ≤ 0.01, ***p ≤ 0.001 compared to LPS and ^#^*p* < 0.05, ^##^*p* ≤ 0.01, ^###^*p* ≤ 0.001 compared to negative control.

For compounds exhibiting divergent effects, namely 2,15-dihydroxycalamenene (**12**), aristelegone A (**14**), and eudesm-4(15)-ene-1*β*,6*β*-diol (**16**), their function as agonists for human TLR4 translated into a consequential agonistic impact on murine TLR4. The ELISA results (Fig. 5a) suggested a moderate reduction in IL-1*β* and a strong inhibition of IL-6 levels by the presence of 10 ng/mL LPS along with stimulations of **12**,** 14**, and **16** at 10 μM in PMA-stimulated THP-1 cells. Simultaneously, the agonist effects were proved by an elevation in the level of IL-1*α* and IL-6 in RAW 264.7 cells (Fig. 5b). Considering the SEAP data (Fig. 2a,b), it becomes apparent that these compounds had lower activity compared to SQ antagonists (**6**,** 9**, and **10**) and showed moderate agonists mTLR4. The TNF-*α* cytokine is a major pathway to activate the NF-κB signaling, as **12**,** 14**, and **16** failed to suppress and recruit the TNF-*α* level, unlike other SQ antagonists and agonists with significant activities observed in HEK cells. In contrast, the expression of IL-1*α* did not significantly contribute to the immunostimulant mTLR4 (SEAP), as their effects on activating TLR4 were relatively similar at concentration of 20 μM (Figs. 2e,f, 5b).

**Figure 6:**
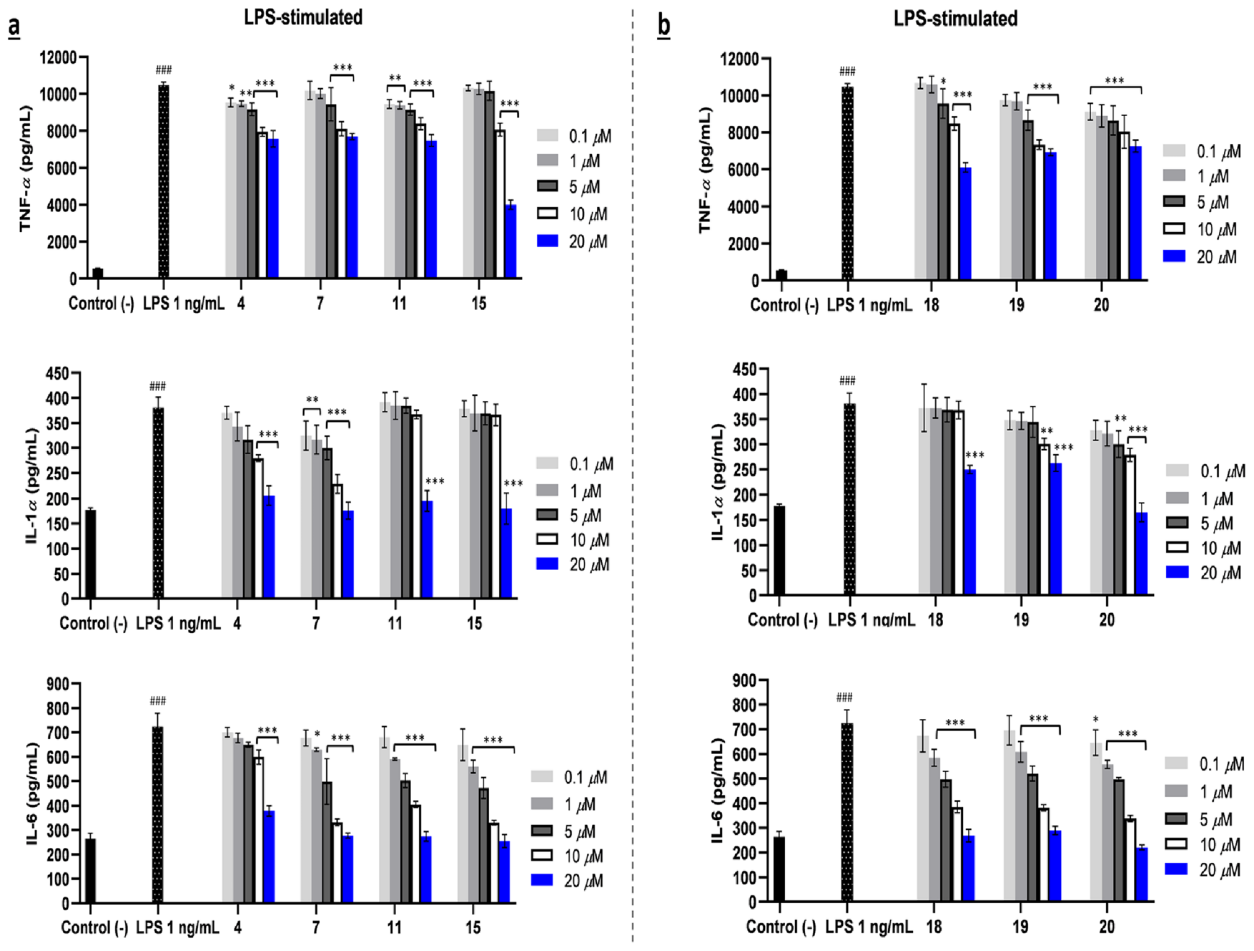
Proinflammatory cytokine productions (TNF-*α*, IL-1*α*, and IL-6) in RAW 264.7 cells as partial antagonist mTLR4 of SQs (**4**, **7**, **11**, and **15**) (**a**) and SQDs (**18–20**) (**b**). The results are shown as means ± SD (n = 4). Significant results are indicated as *p < 0.05, **p ≤ 0.01, ***p ≤ 0.001 compared to LPS and ^#^*p* < 0.05, ^##^*p* ≤ 0.01, ^###^*p* ≤ 0.001 compared to negative control.

The SQs 4*α*,10*α*,11-trihydroxy-1*βH*,5*βH*-guai-7(8)-ene (**4**), 10*β*-hydroxy-4*α*,4*β*-dimethyl-5*αH*,7*αH*-eudesm-3-one (**7**), dysotican B (**11**), and eudesm-4(15)-ene-1*β*,6*α*-diol (**15**) obviously exhibited the immunosuppressive effects on mTLR4 in RAW 264.7 cells at 20 μM across the three proinflammatory used (Fig. 6a). These results indicated that **4**,** 7**,** 11**, and **15** were partial antagonist agents only on murine TLR4 cells. Based on observation, compound **15** showed a two-fold stronger decrease in LPS-stimulated TNF-*α* production compared to others at 20 μM. When referred to the HEK-Blue mTLR4 cells (SEAP) (Fig. 2b), the TLR4 activation of this compound was almost the same as TNF-*α* secretion, including the effect and tendency. Therefore, it was confirmed that TNF-*α* could be the best-identified inducer in murine TLR4, triggering NF-κB regulation. SQDs dysoticans D-F (**18–20**), were also assigned as antagonists due to their effects on decreasing LPS-stimulated expression of cytokines applied in this study (Fig. 6b). Regarding the SEAP (Fig. 2g) and ELISA data (Fig. 6a,b), SQs **7**, **11**, and **15** as well as SQDs **18–20** were partial antagonists exclusively in murine TLR4. This led to an agreement that interactions between hMD-2 and mMD-2 binding regions to the same compound as a ligand were different.

### Structure-activity relationships of SQs and SQDs in their activity on TLR4 receptor

A series of guaiane compounds **1–4** suggested the impacts of several functional groups on their immunomodulatory TLR4 activities. Among these compounds, **4** was the only guaiane variant with activity as immunosuppressive agent against murine cells. These implied that an attachment of hydroperoxide at C-1 of **1** had no effects on its activity since **2** and **3** without hydroperoxide group displayed no activity. The inactive effects of **1–3** compared to **4** also confirmed that hydroxyls at C-4 and C-10 were not crucial on their activity as immunomodulator agents. According to the conclusion from SAR analysis, the Δ^7,8^ in **4** plays a dominant role to its activity within the guaiane series of **1–4**. This assignment was deduced by the presence of 1-hydroperoxide in **1**, 4-OH in **3** and **4**, 7-OH in **1** and **4**, as well as Δ^6,7^ in **2** and **3**. Meanwhile, two series of aromadendrane compounds indicated that the substitution of 14-methyl and hydroxyl at C-10 in **6** enhanced the immunosuppressive effect on the TLR4 receptor. Conversely, the antagonist activity was rendered inactive when these functional groups were replaced by 14-methylene *sp*^*2*^.

**Figure 7:**
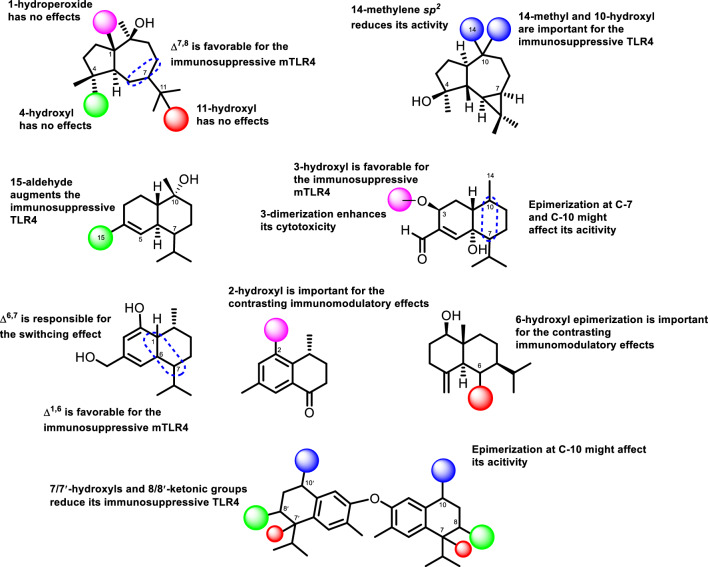
Structure–activity relationship (SAR) of SQs and SQDs.

The existence of 15-aldehyde in a cadinene compound is a key moiety in the structural composition **9**, acting as an antagonist TLR4 agent. This was in contrast to its analog **8** that was inactive due to the occurrence of 15-methyl. Based on the biological activity of compounds **10** and **17**, hydroxyl at C-3 was responsible for the immunosuppressive effect of **10**, while the dimerization strongly increased cytotoxicity against TLR4-based cells. In addition, the epimerization of methyl at C-10 and isopropyl at C-7 in **10** and **17** might be affecting their immunomodulatory activity and cytotoxicity. Similar cadinanes, compounds **11** and **12** by the double bond movement showed that Δ^6,7^ is favorable for partial antagonist against murine TLR4 cells of **11**. On the other hand, Δ^1,6^ is responsible for the switching effect depending on the origin of TLR4 in **12**. The hydroxyl at C-2 in the tris-*nor*-cadinane of **13** showed contrasting effects depending on the origin of the receptor (human or mouse) and become inactive by the hydroxyl shift to C-3 in **14**. The epimerization of hydroxyl at C-6 in 2 eudesmanes, compounds **15** and **16**, extremely altered their immunomodulatory activity. Compound **15** with *α*-hydroxyl acted partial antagonist effect on mTLR4, while *β*-hydroxyl in **16** displayed switching effects in human and murine cells. Consequently, 2 symmetrical dimers **21** and **22** demonstrated that the formation of hydroxyl at C-7ʹ/7ʺ and ketonic at C-8ʹ/8ʺ groups decreased the antagonist activity of **21**. The epimerization of 14-methyl might facilitate the immune regulating effects of these dimers. The SAR of related SQs and SQDs was presented in Fig. 7.

## Conclusions

In conclusion, this study evaluated the immunomodulatory activity of 16 SQs and six SQDs previously isolated from *D. parasiticum* (Osbeck). Kosterm stem bark on TLR4 receptor of human and murine cells. Based on the results, SQs **6**,** 9**, and **10** and SQDs **17** and **22** were classified as antagonist TLR4 on both cells. Among these compounds, 10-hydroxyl-15-oxo-*α*-cadinol (**9**) was discovered to be the most potent anti-inflammatory agent without negative effects on cell viability. On the other hand, several compounds, including SQs **4**,** 7**,** 11**, and **15** as well as SQDs **18**,** 19**, and **22**, regulated partial antagonist exclusively on murine TLR4 cells. A switching effect was observed during the transition from human to murine TLR4/MD-2/CD14 system with SQs **12**,** 14**, and **16** moving from antagonistic hTLR4 to agonistic mTLR4. Furthermore, a brief structure-activity relationship presented the impacts of double bond position, attachment of hydroxyl, aldehyde, and methyl groups, as well as position of hydroxyl and epimerization, which are crucial to the immune regulating system on TLR4 receptor.

## Materials and methods

### Extract preparation and isolation of SQs and SQDs

*D. parasiticum* (Osbeck) Kosterm. was collected from Bogor Herbarium Botanical Garden (BHBG), West Java Province, Indonesia (latitude -6.597629, longitude 106.799568, elevation 277 m), where it is widely cultivated. Identification and authentification of the plant were conducted at the BHGB by Mr. Harto, an expert in the BHGB, and a voucher specimen (III. F.79) was deposited for the plant species. Collection and experimental research on this plant comply with relevant institutional, national, and international guidelines and legislation.

The dried stem bark was powdered and extracted with MeOH. Subsequently, 16 SQs and 6 SQDs were isolated from the *n-*hexane and ethyl acetate extracts by chromatography over silica gel, followed by purification on reverse phase ODS, as described in previous publications^22–24^.

### SEAP NF-κB-dependent reporter assay

HEK-Blue cells (hTLR4 and mTLR4, InvivoGen) were cultured in accordance with the instructions of the manufacturer. The cells were cultured in Dulbecco’s modified Eagle’s medium (DMEM) high glucose supplemented with 1% penicillin-streptomycin and 10% fetal bovine serum (FBS). A 96-well multiwell plate was seeded with a total of 2 ×  10^4^ cells/well in medium supplemented with 1% penicillin-streptomycin. The plate was incubated at 37 ℃, 5% CO_2_, and 95% humidity overnight. Subsequently, the supernatants were replaced by fresh DMEM high glucose (supplemented with 0.1% FBS and 1% penicillin-streptomycin). Samples were added at different concentrations (0.1; 1; 5; 10; and 20 μM) and exposed to 10 ng/mL and 1 ng/mL of LPS from *E. coli* (Fujifilm Wako Pure Chemical Co., Ltd) for hTLR4 and mTLR4, respectively, up to 24 h. Additionally, supernatants containing SEAP were gathered and combined with *para*-nitrophenyl phosphate (*p*NPP) at room temperature for 4 h. A microplate reader was used to measure the optical density (OD) at 405 nm.

### Sandwich ELISA assay

THP-1 cells (JCRB0112) were purchased form the JCRB Cell Bank (Ibaraki, Osaka, Japan) and cultured in Roswell Park Memorial Institute (RPMI) 1640 (+ 10% FBS and + 1% penicillin-streptomycin). The cells were used at a density of 1 × 10^5^ cells/well and differentiated into macrophages by adding 0.3 *μ*L/mL phorbol 12-myristate 13-acetate (PMA) for 24 h. After incubation, the medium was replaced by new RPMI (+ 1% FBS, + 1% penicillin–streptomycin, and + 10 mM HEPES) and incubated for 24 h. The cells were pretreated with all compound variants (0.1–20 μM) for 1 h, followed by exposure to LPS (10 ng/mL) up to 24 h for LPS-stimulated design.

RAW 264.7 cells (EC91062702-F0) were purchased from the Kyorin Pharmaceutical Group Factories Sumitomo Pharma Co., Ltd, Japan and cultured in DMEM high glucose, supplemented with 10% FBS and 1% penicillin-streptomycin. Cell scraper was used to detach cells, and the cell concentration was estimated by Trypan Blue (Sigma-Aldrich). Furthermore, a density of 1 × 10^4^ cells/well was seeded into 96-well plates in fresh DMEM high glucose (1% FBS, + 1% penicillin–streptomycin, and + 10 mM HEPES). Following overnight incubation (37 ℃, 5% CO2, 95% humidity), the cells were treated with increasing concentrations of samples dissolved in EtOH and diluted in DMEM. After 1 h, cells were stimulated with 10 ng/mL of LPS for up to 24 h.

For the determination of cytokine production, supernatants of THP-1 and RAW 264.7 were collected and tested using sandwich enzyme-linked immunosorbent assay (ELISA). The proinflammatory cytokines of TNF-α (LOT: 218819-004, Invitrogen), IL-1β (LOT: 252921-005, Invitrogen), and IL-6 (LOT: 23638-011, Invitrogen) from human uncoated ELISA kit were adopted for THP-1 supernatant. Meanwhile, the RAW 264.7 supernatant was analyzed using murine uncoated ELISA kit of TNF-α (LOT: 306473-002, Invitrogen), IL-1α (LOT: B352593, Biolegend), and IL-6 (309364-001, Invitrogen). The assay was conducted according to the specification of the manufacturer, including the coating of capture antibody, blocking using ELISPOT, adding the collected supernatant, and preparation of the standard of protein of interest. The measurement of targeted proinflammatory cytokines was performed by adding the detection antibody, enzyme of horseradish peroxidase (HRP), and substrate of tetramethylbenzidine (TMB). Additionally, the OD was observed on a microplate reader at 450 nm.

### Viability assay

Cell viability was assessed after supernatant collection. The monolayers of remaining cells (HEK-Blue hTLR4, HEK-Blue mTLR4, THP-1, and RAW 264.7) in the 96-well plates were added with 90 *μ*L/well of new medium and 10 *μ*L/well of cell counting kit 8 (CCK8). Additionally, the formazan concentration was determined by measuring the OD at 450 nm on a microplate reader.

### Statistical analysis

All experiments were performed independently in 4 replicates. The results were normalized with control cells which were not exposed to any drug. The GraphPad Prism 8 software (GraphPad Software, San Diego, CA, USA) was used to generate the statistical analyses. In addition, each data was visualized as the mean ± standard deviation (SD). The one-way analysis of variance (ANOVA) and Dunnett’s or Bonferroni multiple comparison post-tests were adopted for significant comparison between treatment groups. A *p-*value < 0.05 was considered statistically significant (*p < 0.05, **p ≤ 0.01, ***p ≤ 0.001 compared to LPS and ^#^*p* < 0.05, ^##^*p* ≤ 0.01, ^###^*p* ≤ 0.001 compared to negative control).

### Supplementary Information

Supplementary Information.

## Data Availability

All data generated or analysed during this study are included in this published article and its supplementary information files.

## Abbreviations

SQs: Sesquiterpenoids
SQDs: Sesquiterpenoid dimers
TLR4: Toll-like receptor 4
hTLR4: Human TLR4
mTLR4: Murine TLR4
LPS: Lipopolysaccharide
LBP: LPS-binding protein
LOS: Lipopolysaccharide
CD14: Cluster to differentiation 14
MD-2: Myeloid differentiation factor 2
SEAP: Secreted alkaline phosphatase
NF-κB: Nuclear factor kappa-light-chain-enhancer of activated B
THP-1: Tohoku hospital pediatrics-1
RAW 264.7: Ralph, raschke, Watson 264.7
TNF-α: Tumor necrosis factor
IL-1/6: Interleukin-1/6
ASC: Apoptosis-associated speck-like protein
ELISA: Enzyme-linked immunosorbent assay
*p*NPP: *para*-Nitrophenyl phosphate
RPMI: Roswell park memorial institute
DMEM: Dulbecco’s modified eagle’s medium
FBS: Fetal bovine serum
HRP: Horseradish peroxide
TMB: Tetramethylbenzidine
OD: Optical density
CCK8: Cell counting kit 8
