# Supplementary material for: Immunomodulatory of sesquiterpenoids and sesquiterpenoid dimers-based toll-like receptor 4 (TLR4) from Dysoxylum parasiticum stem bark
Source: Sci Rep. 2024 Jul 6;14:15597. doi: 10.1038/s41598-024-65829-0 (PMC11227493; doi:10.1038/s41598-024-65829-0)
Supplement: Supplementary file 1 — Supplementary Information. [file 41598_2024_65829_MOESM1_ESM.docx]

**Supporting Information**

**Immunomodulatory of sesquiterpenoids and sesquiterpenoid dimers-based toll-like receptor 4 (TLR4) from *Dysoxylum parasiticum* stem Bark**

Al Arofatus Naini^1,2^, Tri Mayanti^1,3^, Erina Hilmayanti^4^, Xuhao Huang^4^, Kazuya Kabayama^5^, Atsushi Shimoyama^4^, Yoshiyuki Manabe^4^, Koichi Fukase^4^, Unang Supratman^1,2*^

^1^Department of Chemistry, Faculty of Mathematics and Natural Sciences, Universitas Padjadjaran, Jatinangor 45363, Sumedang, West Java, Indonesia

^2^Central Laboratory, Universitas Padjadjaran, Jatinangor 45363, Sumedang, West Java, Indonesia

^3^Study Centre of Natural Product Chemistry and Synthesis, Faculty of Mathematics and Natural Sciences, Universitas Padjadjaran, Jatinangor 45363, Sumedang, West Java, Indonesia

^4^Department of Chemistry, Graduate School of Science, Osaka University, 1-1 Machikaneyama-cho, Toyonaka, Osaka, 560-0043, Japan

^5^Institute for Radiation Sciences, Osaka University, 1-1 Machikaneyama-cho, Toyonaka, Osaka, 560-0043, Japan

^*^Corresponding author. Tel./Fax: +62-22-7794391;

E-mail: unang.supratman@unpad.ac.id

**Contents**

[Figure S.1 Optimization of LPS against HEK-Blue hTLR4 and mTLR4 3](#_Toc153840289)

[Figure S.2 MTT assay of selected compounds against HEK-Blue hTLR4 3](#_Toc153840290)

[Figure S.3 MTT assay of selected compounds against HEK-Blue mTLR4 4](#_Toc153840291)

[Figure S.4 MTT assay of selected compounds against THP-1 4](#_Toc153840292)

[Figure S.5 MTT assay of selected compounds against RAW 264.7 5](#_Toc153840293)


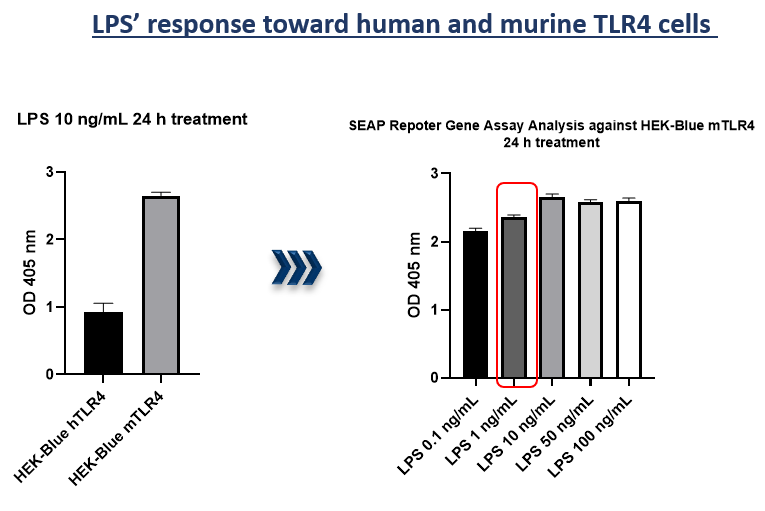


Figure S.1 Optimization of LPS against HEK-Blue hTLR4 and mTLR4

Figure S.2 MTT assay of selected compounds against HEK-Blue hTLR4

Figure S.3 MTT assay of selected compounds against HEK-Blue mTLR4

Figure S.4 MTT assay of selected compounds against THP-1

Figure S.5 MTT assay of selected compounds against RAW 264.7
